# Mechanism Underlying *Ganoderma lucidum* Polysaccharide Biosynthesis Regulation by the *β*-1,3-Glucosyltransferase Gene *gl20535*

**DOI:** 10.3390/jof11070532

**Published:** 2025-07-17

**Authors:** Jingyun Liu, Mengmeng Xu, Mengye Shen, Junxun Li, Lei Chen, Zhenghua Gu, Guiyang Shi, Zhongyang Ding

**Affiliations:** 1 School of Biotechnology and Key Laboratory of Carbohydrate Chemistry and Biotechnology of Ministry of Education, Jiangnan University, Wuxi 214122, China; 7180201011@stu.jiangnan.edu.cn (J.L.); xmm900801@163.com (M.X.); 7170201015@stu.jiangnan.edu.cn (M.S.); leichen@jiangnan.edu.cn (L.C.); guzhenghua2011@163.com (Z.G.); gyshi@jiangnan.edu.cn (G.S.); 2 National Engineering Research Center for Cereal Fermentation and Food Biomanufacturing, Jiangnan University, Wuxi 214122, China; 3 School of Food Science and Technology, Jiangnan University, Wuxi 214122, China; 4 Shandong Taishan Shengliyuan Group Co., Ltd., Taian 271000, China

**Keywords:** *Ganoderma lucidum*, *β*-1,3-glucosyltransferase, polysaccharide synthesis

## Abstract

*Ganoderma lucidum* polysaccharides (GLPs) are natural compounds with a broad spectrum of biological activities. *β*-1,3-glucosyltransferase (GL20535) plays an important role in polysaccharide synthesis by catalyzing the transfer of UDP-glucose to extend sugar chains, but its underlying mechanism remains unclear. In this study, the regulatory mechanism of GL20535 in polysaccharide synthesis was elucidated by overexpressing and silencing *gl20535* in *G. lucidum*. Overexpression of *gl20535* resulted in maximum increases of 18.08%, 79.04%, and 18.01% in intracellular polysaccharide (IPS), extracellular polysaccharide (EPS), and *β*-1,3-glucan contents, respectively. In contrast, silencing *gl20535* resulted in maximum reductions of 16.97%, 30.20%, and 23.56% in IPS, EPS, and *β*-1,3-glucan contents, respectively. These phenomena in the overexpression strains were attributed to *gl20535*-mediated promotion of UDP-glucose synthesis in the sugar donor pathway and upregulation of the expression of glycoside hydrolase genes. The opposite trend was observed in the silenced strains. In mycelial growth studies, neither overexpression nor silencing of *gl20535* affected biomass and cell wall thickness. Furthermore, the GL20535 isozyme gene *gl24465* remained unaffected in *gl20535*-overexpressed strains but was upregulated in *gl20535*-silenced strains, suggesting a compensatory regulatory relationship. These findings reveal the regulatory role of GL20535 on gene expression in the GLPs synthesis pathway and deepen our understanding of GL20535 function in the polysaccharide network of edible and medicinal fungi.

## 1. Introduction

*Ganoderma lucidum*, a renowned edible and medicinal fungus [1,2], exhibits a wide range of pharmacological properties, including immunoregulatory, anti-inflammatory and prebiotic activities [3,4]. *G. lucidum* polysaccharides (GLPs) are typically heteropolysaccharides composed of various monosaccharides linked by *β*-1,3-, *β*-1,6-, *α*-1,3- and *α*-1,4-glycosidic bonds [5]. The GLP structures are predominantly composed of *β*-glucan, which is characterized by a main chain of *β*-1,3-linked glucose units with *β*-1,6-linked side chains [6].

GLPs biosynthesis is a complex metabolic regulatory network involving the synthesis of nucleotide sugar donors, extension of the sugar chain, and export of polysaccharides [7]. With the advancement in metabolic modeling, the key enzymes involved in these pathways have gradually been elucidated [8]. In the initial sugar donor synthesis process, overexpression of phosphoglucomutase (PGM) and UDP-glucose pyrophosphorylase (UGP) enhances UDP-glucose synthesis, thereby increasing both polysaccharide yield and glucose content [9,10]. Similarly, phosphomannose isomerase (PMM), phosphomannomutase (PMI), and GDP-mannose pyrophosphorylase (GMP) are involved in GDP-mannose biosynthesis, affecting the mannose composition of polysaccharides [11,12]. These enzymes not only redirect carbon flux toward sugar donor biosynthesis but also influence the monosaccharide composition of GLPs [7].

In the subsequent process, sugar donors are transferred to acceptor molecules via specific glycosidic linkages, forming structurally defined polysaccharide chains [13,14]. Glycosyltransferases (GTs) play a pivotal role in this process [15]. For instance, *α*-1,3-glucosyltransferase [16] and *β*-1,3-glucosyltransferase [17], along with UGT88A1 [18], have been implicated in mushroom polysaccharide biosynthesis. In *Grifola frondosa*, silencing genes encoding *β*-1,3-glucosyltransferases, particularly *GFGLS2*, inhibited both mycelial growth and polysaccharide synthesis [19]. Comparatively, in *G. lucidum*, *β*-1,3-glucosyltransferases exhibited elevated expression in high polysaccharide-producing strains [20]; overexpression of genes encoding these transferases in *G. lingzhi* increased intracellular polysaccharide (IPS) content; and co-overexpression with *ugp* further enhanced total polysaccharide yield [21,22]. Although existing studies have highlighted a correlation between *β*-1,3-glucosyltransferase expression and polysaccharide production in *Ganoderma*, the precise functions and regulatory mechanisms of these genes remain unclear.

In this study, *G. lucidum* CGMCC 5.26 was employed to investigate the functional role of *β*-1,3-glucosyltransferase (GL20535) in polysaccharide biosynthesis. Overexpression and silencing strains were constructed to assess the effects of GL20535 on mycelial growth, morphology, and polysaccharide production. Furthermore, transcriptional profiling of key biosynthetic genes was performed to elucidate the underlying regulatory mechanisms. The findings could provide a deeper insight into the role of *β*-1,3-glucosyltransferase in *G. lucidum* and a theoretical foundation for further refining the polysaccharide biosynthesis pathway.

## 2. Materials and Methods

### 2.1. Strain and Culture Medium

The wild-type (WT) strain *G. lucidum* CGMCC 5.26 and recombinant strains were preserved on potato dextrose agar at 4 °C and incubated at 30 °C. For submerged culture, homogenized seed culture (300 mg wet-weight) was inoculated into 80 mL of liquid fermentation medium (containing 20 g/L glucose, 5 g/L tryptone, 5 g/L yeast nitrogen base without amino acids, 4.5 g/L KH_2_PO_4_, and 2 g/L MgSO_4_·7H_2_O) and cultivated at 30 °C with agitation at 150 rpm for 10 d. Transformants were screened on casein yeast extract medium (composed of 20 g/L glucose, 10 g/L maltose, 2 g/L peptone, 2 g/L yeast extract, 4.6 g/L KH_2_PO_4_, and 0.5 g/L MgSO_4_·7H_2_O). For plasmid construction, *Escherichia coli* JM109 served as the cloning host.

### 2.2. Cloning and Bioinformatic Analysis of gl20535

The *β*-1,3-glucosyltransferase gene *gl20535* (Gene bank: AZQ26797.1) [23] was amplified from *G. lucidum* genomic DNA (gDNA) and complementary DNA (cDNA) using the primer pairs *gl20535*-F/R (Table S1). Briefly, gDNA was extracted using the CTAB method [24]. The total RNA was extracted from 6-day-old mycelial cultures using FastPure^®^ Universal Plant Total RNA Isolation Kit (Vazyme, Nanjing, China). The cDNA template was generated by reverse transcription of total RNA using HiScript^®^III RT SuperMix for qPCR (+gDNA wiper) (Vazyme, Nanjing, China), following the manufacturer’s protocol. The identity of all cloned gene sequences was confirmed by sequencing (Sangon Biotech, Shanghai, China).

The deduced amino acid sequence of *gl20535* was subjected to bioinformatic analyses [25]. The NCBI Conserved Domain database (https://www.ncbi.nlm.nih.gov/) was used for domain prediction, while sequence homology and phylogeny of fungal *β*-1,3-glucosyltransferases were assessed via NCBI Protein BLAST (https://blast.ncbi.nlm.nih.gov/Blast.cgi). A phylogenetic tree was constructed using MEGA 6.06 and visualized in iTOL (https://itol.embl.de/itol.cgi). Molecular weight and isoelectric point (pI) were computed using ProtParam (https://web.expasy.org/protparam/), while hydrophobicity was analyzed using ProtScale (https://web.expasy.org/protscale/). Signal peptides and transmembrane domains were predicted using SignalP 6.0 and TMHMM (http://www.cbs.dtu.dk/services/TMHMM/), respectively.

### 2.3. Construction of the Overexpression and Silenced Plasmids

Following established plasmid manipulation strategies [16], the carboxin-resistance plasmids pMsdhB-OE*gl20535* and pMsdhB-i*gl20535* were constructed (Figure S1) using the primers listed in Table S1. Mutant *glsdhB* (MsdhB) was cloned into pMD19-T to construct pMsdhB. Two cassettes were then ligated to pMsdhB: an overexpression cassette containing the P*glgpd* promoter and T*glsdhB* terminator, yielding pMsdhB-OEGL, and a silencing cassette including P*glgpd*, URA3-silenced sequence (*ura3*), and the CaMV35s promoter (P*35s*), producing pMsdhB-RNAiGL. The *gl20535* cDNA was inserted into pMsdhB-OEGL to generate the final overexpression plasmid, pMsdhB-OE*gl20535*, while a conserved *gl20535* fragment was ligated into pMsdhB-RNAiGL to create the silencing plasmid, pMsdhB-i*gl20535*. All cloning steps were performed using the ClonExpress^®^ Ultra One-Step Cloning Kit (Vazyme, Nanjing, China).

### 2.4. Construction and Identification of Overexpression and Silenced Strains

Transformants were generated using the PEG-mediated protoplast transformation (PMT) method [16]. Protoplasts were prepared from 4-day-old static cultures by suspending the harvested mycelia in 0.6 M mannitol containing 20% (*w/v*) lysing enzymes (Guangdong Microbiology Culture Center, Guangzhou, China), followed by enzymatic digestion at 30 °C and 150 rpm for 2.5 h. After filtration, 10 μg of plasmid DNA were added to 167 μL STC buffer (0.55 M sorbitol, 10 mM CaCl_2_, 10 mM Tris-HCl, pH 7.5) containing approximately 10^7^ protoplasts/mL, along with 5 μL spermidine and 2 μL heparin. The mixture was incubated on ice for 10 min before sequential addition of 400 μL and then 600 μL PTC buffer (40% PEG4000, 50 mM CaCl_2_, 10 mM Tris-HCl, pH 7.5), followed by incubation at 28 °C for 30 min. The protoplasts were collected by centrifugation (3500× *g*, 10 min, 28 °C), resuspended in 1 mL CYM regeneration medium, and plated on CYM agar. After 2 days at 30 °C, 4 μg/mL carboxin in soft agar was overlaid to select transformants, which were further incubated for 10–14 days.

In addition, control (pMsdhB), overexpression (pMsdhB-OE*gl20535*), and RNAi silencing (pMsdhB-i*gl20535*) plasmids were independently transfected into *G. lucidum* protoplasts, and the transformants were selected on CYM agar medium containing 4 mg/L carboxin. The overexpression strains were verified by amplifying the expression cassette from gDNA using P*gpd*-F/*sdhB*-R primers (Table S1), with sequence-confirmed transformants designated as Oe-*gl20535*. Similarly, silenced strains were authenticated using the P*gpd*-F/P*35s*-R primers (Table S1) and named i*gl20535* after sequence verification, while the control transformants retained the control designation.

### 2.5. Real-Time Quantitative PCR (RT-qPCR) Analysis of Gene Expression

RT-qPCR analysis was carried out using a ChamQ Universal SYBR qPCR Master Mix (Vazyme, Nanjing, China) following established protocols, and the 18S rRNA gene was used as an internal control for normalization [16]. Gene expression levels were calculated using the 2^−ΔΔCt^ comparative method [26]. Briefly, the threshold cycle (Ct) of each target gene was first normalized to that of the internal reference gene ΔCt (ΔCt = Ct_target_ − Ct_reference_) and then compared to the ΔCt of the control group to calculate ΔΔCt (ΔΔCt = ΔCt_sample_ − ΔCt_control_). The fold change in gene expression was determined using the formula 2^−ΔΔCt^.

Transcript levels were measured across WT, Control, Oe-*gl20535*, and i*gl20535* strains for key metabolic genes, including *pgm*, *ugp*, the UDP-glucose 4-epimerase gene (*uge*), the phosphoglucose isomerase gene (*pgi*), *pmi*, *pmm*, *gmp*, *β*-1,3-glucosyltransferases (*gl20535* and *gl24465*), *β*-1,3-glucanohydrolases (*gl24581*, *gl21451*, *gl27365*, *gl30087*, and *gl20743*), and chitin synthases (*gl15273*, *gl25613*, *gl28060*, *gl30799*, and *gl31550*). The primer sequences are detailed in Table S2.

### 2.6. Characterization of Mycelial Morphology

After a 6-day cultivation at 30 °C in both liquid and plate media, mycelial morphology of WT and recombinant strains was examined using a Nikon SMZ25 camera. Colony radii and pellet diameters were quantified using ImageJ software v1.8.0 [27]. Mycelial pellet size was classified into S-fraction (diameter < 1 mm), M-fraction (1 mm < diameter < 2 mm), and L-fraction (diameter > 2 mm).

Mycelia from the liquid culture were collected, rinsed, and postfixed in 2.5% (*v/v*) glutaraldehyde for ultrathin sectioning. The ultrastructure of the mycelial cross-sections was subsequently examined using a transmission electron microscope (TEM, Hitachi HT-7800, Tokyo, Japan). Mycelial diameter and cell wall thickness were measured using the ImageJ software.

### 2.7. Measurements of Biomass, Residual Sugars, EPS, and IPS

Biomass, residual sugar, and polysaccharides were analyzed as previously described [27]. The mycelia were centrifuged, washed, and freeze-dried for dry weight measurements. IPS was extracted from the dried powder via hot-water extraction, while EPS and residual sugars were measured from the collected fermentation broth. IPS and EPS were ethanol-precipitated and quantified using the phenol–sulfuric acid method [28]. Residual sugars were determined using the DNS method [29].

### 2.8. Comparison of Monosaccharides

The monosaccharide composition of the polysaccharides was analyzed using ion chromatography [16]. Monosaccharides were identified by comparing their retention times, and their proportions were determined through content percentage (Figure S2).

### 2.9. Quantification of β-1,3-glucan and Chitin in the Cell Wall

The freeze-dried mycelium powder was pulverized and sequentially washed with aqueous solutions of NaCl (1%, 2%, and 5%), followed by eight ddH_2_O washes. The purified cell wall powder was freeze-dried and crushed [30].

The *β*-1,3-glucan in the cell wall was alkali-extracted (1 M NaOH) and quantified fluorometrically using aniline blue staining [27]. Fluorescence intensity was detected using a microplate reader (Hitachi F-7000) at 405 nm excitation and 460 nm emission wavelengths. *β*-1,3-glucan content (%) was quantified as fluorescence intensity per milligram of cell wall and expressed in relative units [16,31].

Chitin content was determined by measuring the glucosamine levels in the cell wall after complete enzymatic hydrolysis, following the method described by Popolo et al. [32] and Chen et al. [16]. Chitin content (%) was quantified as glucosamine content per milligram of cell wall and expressed in relative units.

### 2.10. Statistical Analysis

GraphPad Prism 9.5 was used for statistical analysis. Data from triplicate experiments are presented as mean ± SD (n = 3). Significant differences (* *p* < 0.05, ** *p* < 0.01) were determined by one-way ANOVA with Duncan’s multiple range test.

## 3. Results

### 3.1. Silencing and Overexpression of gl20535

**Figure 1:**
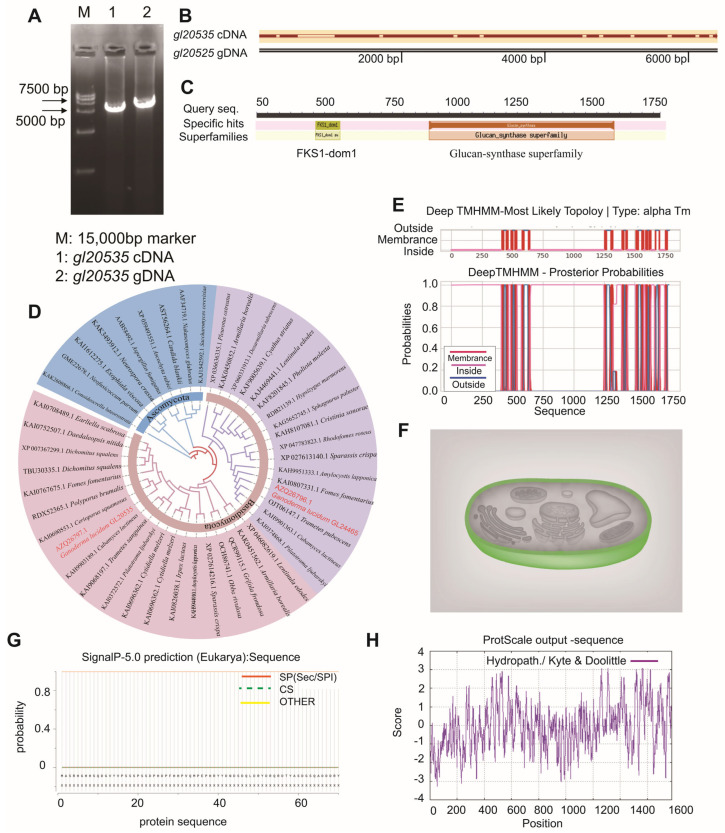
Bioinformatics analysis of the *β*-1,3-glucosyltransferase gene *gl20535*. (**A**) Cloning of *gl20535* cDNA and gDNA sequences from G. lucidum; (**B**) sequence comparison of *gl20535* cDNA and gDNA, indicating exon–intron structure; (**C**) conserved domain analysis using the NCBI Conserved Domain Database; the protein harbors a *β*-1,3-glucan synthase subunit FKS1 homolog domain (fks-dom1) and *β*-1,3-glucan synthase component domain (glucan synthase); (**D**) phylogenetic tree of *β*-1,3-glucosyltransferases from G. lucidum and related fungi; (**E**) transmembrane domain prediction using TMHMM v2.0 with red bars indicating the locations of 17 predicted transmembrane helices; (**F**) subcellular localization prediction: colors indicate predicted compartments, with green representing cytoplasmic localization; (**G**) hydropathy plot generated using ProtScale; higher values indicate more hydrophilic regions, while lower values correspond to more hydrophobic segments of the protein; (**H**) signal peptide prediction using SignalP: no signal peptide was predicted.

The gDNA sequencing of the *β*-1,3-glucosyltransferase gene *gl20535* from *G. lucidum* revealed a full-length sequence of 6408 bp (Figure 1A). A comparison with its cDNA sequence indicated the presence of 11 introns, with a total exon length of 5343 bp, encoding a 1780-amino acid polypeptide (Figure 1A,B). Bioinformatic predictions estimated the molecular weight of the encoded protein to be 204.15 kDa, with an isoelectric point (pI) of 9.14. The protein was characterized as a basic, hydrophilic, membrane-associated protein lacking signal peptides and containing 17 transmembrane helices (Figure 1E–H). Conserved domain analysis identified both a *β*-1,3-glucan synthase subunit FKS1 homologous domain (fks1-dom1) and a *β*-1,3-glucan synthase component domain (glucan synthase) (Figure 1C), which are recognized as essential catalytic regions in fungal *β*-1,3-glucan synthases [33,34,35]. Additionally, multiple sequence alignment showed that *gl20535* shares high sequence similarity with *β*-1,3-glucosyltransferase genes from *Cerioporus squamosus* (KAI0690553.1), *Grifola frondosa* (QCR99115.1), *Lentinula edodes* (XP046082619.1), and other fungi (Figure 1D).

**Figure 2:**
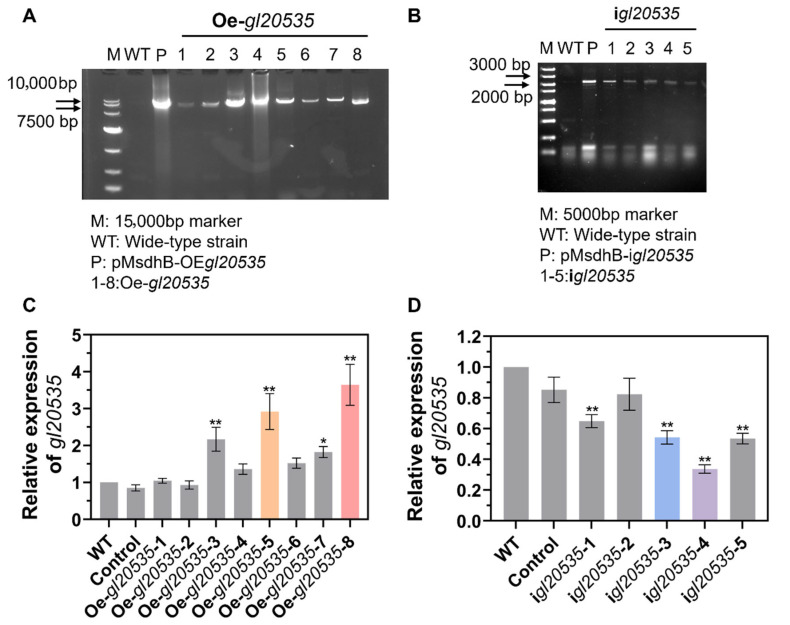
Construction of *gl20535* overexpression and silenced strains. PCR confirmation of (**A**) overexpression strains Oe-*gl20535*-n (n = 1–8) and (**B**) silenced strains i*gl20535*-n (n = 1–5) using plasmid-specific primers. The transcription levels of *gl20535* in (**C**) overexpression strains and (**D**) silenced strains compared to those in the wild type (WT). Results are expressed as mean ± standard deviation from triplicate experiments (n = 3); * (*p* < 0.05) and ** (*p* < 0.01) indicate significant differences compared to the WT.

To further analyze the function of GL20535 in *G. lucidum*, overexpression and silencing strains were constructed using the PMT method. Based on bioinformatics analysis, the fks1-dom1 domain (amino acid residues 261 to 372) in the *gl20535* cDNA sequence was selected as the target for constructing the silenced strain i*gl20535* (Figure 1C). Simultaneously, *gl20535* cDNA was overexpressed in *G. lucidum* to generate the overexpression strain Oe*-gl20535*. Following carboxin resistance screening and PCR verification, eight transformants were confirmed to have successfully integrated the overexpression plasmid (Figure 2A), while five transformants were verified to have successfully incorporated the silencing plasmid (Figure 2B).

RT-qPCR was performed to evaluate the transcript level of *gl20535* in the recombinant strains. The results indicated no significant differences between the control and WT strains, suggesting that molecular manipulation did not affect the expression of *gl20535* in *G. lucidum*. Among the transformants, strains Oe-*gl20535*-5 and Oe-*gl20535*-8 showed significant upregulation, with expression levels reaching 292.00% and 364.27% of the WT, respectively (Figure 2C). Conversely, strains i*gl20535*-3 and i*gl20535*-4 exhibited a reduction in *gl20535* transcript levels by 48.01% and 66.32%, respectively, compared to the WT strain (Figure 2D). Therefore, four recombinant strains (i*gl20535*-3, i*gl20535*-4, Oe-*gl20535*-5, and Oe-*gl20535*-8) were selected for further analysis.

### 3.2. Effect of GL20535 on Mycelial Morphology and Growth Characteristics

#### 3.2.1. Morphological Characteristics

**Figure 3:**
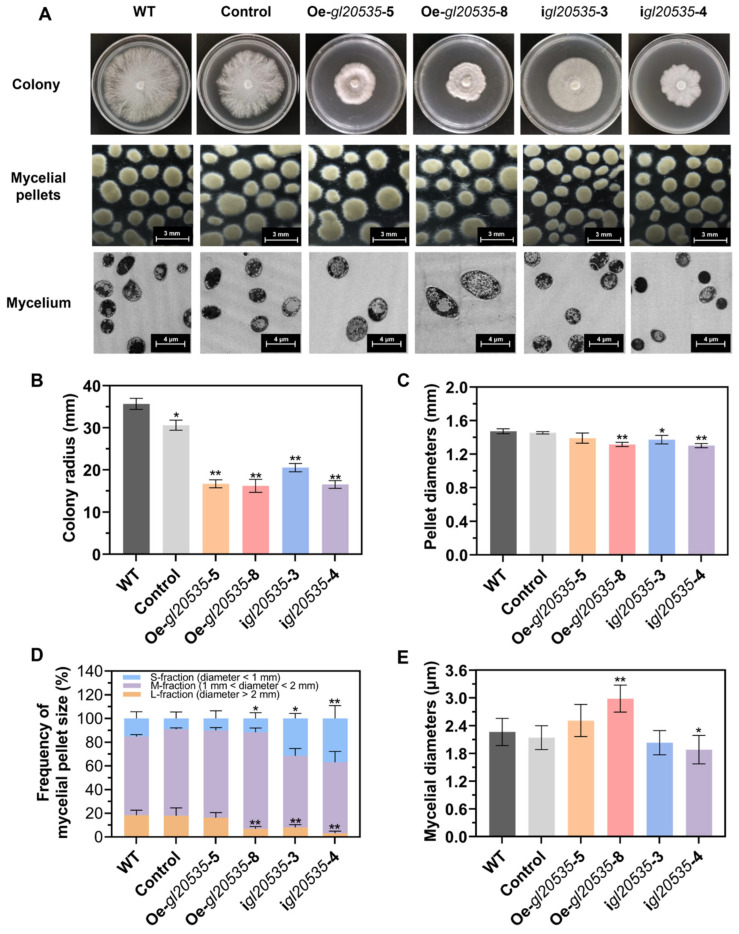
Effect of GL20535 on morphological characteristics. (**A**) Macro-morphological analysis of the WT and recombinant strains after 6 d of culture in both plate and liquid media. (**B**) The mean colony radius of recombinant strains in plate culture. (**C**) The mean pellet diameters of recombinant strains in liquid culture. (**D**) The mycelial pellet size classified by diameter: S, M, L, and their calculated proportions. (**E**) The mean mycelial diameters of recombinant strains in liquid culture. Results are expressed as mean ± standard deviation from triplicate experiments (n = 3); * (*p* < 0.05) and ** (*p* < 0.01) indicate significant differences compared to the WT.

On a solid medium, *gl20535* expression inhibited mycelial growth and induced a distinct colony morphology (Figure 3A). The WT strain exhibited radial and uniform growth, characterized by sparse, fluffy hyphae, and smooth colony edges, with a mean colony radius of 35.7 ± 1.1 mm. The colony radius of the Oe-*gl20535*-5, Oe-*gl20535*-8, i*gl20535*-3, and i*gl20535*-4 strains were significantly smaller, with reductions of 55.65%, 56.48%, 42.47%, and 55.05%, respectively (*p* < 0.05), compared with those of the WT strain (Figure 3B). In addition, the control strain also exhibited a decrease in colony radius compared to the WT, indicating that genetic manipulation affected mycelial growth on solid medium.

In submerged cultures, the morphological characteristics of mycelial pellets, particularly diameter and surface roughness, serve as indicators of mycelial growth and polysaccharide production [36]. In the WT strain, the mean pellet diameter was 1.47 ± 0.02 mm (Figure 3C), with M-fraction pellets (1 mm < diameter < 2 mm) being predominant, accounting for 66.37% (Figure 3D). Overexpression of *gl20535* reduced the pellet diameter to 1.33 ± 0.01–1.39 ± 0.05 mm, accompanied by an increase in the percentage of M-fraction mycelia to 73.88–81.65% (Figure 3C,D). Morphologically, *gl20535* overexpression strains exhibited ovoid and starburst-shaped pellets (Figure 3A). Conversely, the M-fraction pellets of *gl20535*-silenced strains showed no significant change (*p* > 0.05), but the S-fraction pellets (diameter < 1 mm) significantly increased to 31.47–36.90% (*p* < 0.05) (Figure 3D) and the diameter of the pellets decreased to 1.30 ± 0.02–1.37 ± 0.04 mm (Figure 3C). The mycelial morphology of the silenced strains changed to small and smooth. Although the pellet diameter of recombinant strains was reduced, *gl20535* overexpression strains, with uniform and hairy pellets may be linked to the liberation of the second generation of pellet, while the formation of small and bead-like pellets in *gl20535*-silenced strains inhibited mycelium aggregation [37]. When observed by TEM, the average mycelial diameter of the WT strain was 2.26 ± 0.29 μm. In *gl20535* overexpression strains, the mycelial diameter increased by 10.93–31.87% compared to that in the WT, with thicker mycelia observed in strains exhibiting higher *gl20535* expression levels. Conversely, in *gl20535*-silenced strains, the mycelial diameter decreased by 10.25–16.89%, indicating thinning of the mycelium (Figure 3E).

#### 3.2.2. Growth Curve

**Figure 4:**
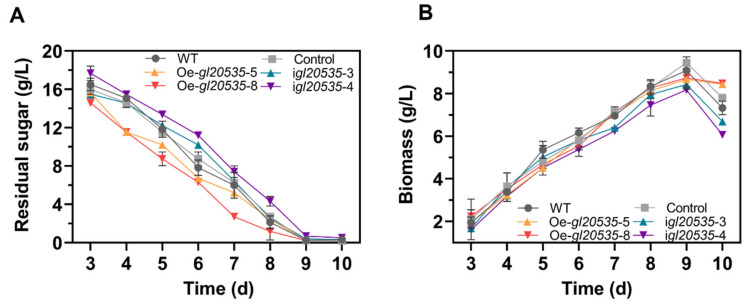
Effect of GL20535 on growth in submerged culture. (**A**) Residual sugar. (**B**) Biomass. Results are expressed as mean ± standard deviation from triplicate experiments (n = 3).

Glucose consumption and biomass accumulation during submerged fermentation by the WT and recombinant strains are shown in Figure 4. Overexpression of *gl20535* accelerated glucose consumption but had no effect on biomass accumulation. Strains Oe-*gl20535*-5 and Oe-*gl20535*-8 reached maximum biomass on day 9, with values of 8.67 ± 0.11 g/L and 8.73 ± 0.10 g/L, representing a slight reduction of 3.79% and 4.57%, respectively, compared to those in the WT strain (9.08 ± 0.10 g/L). In contrast, *gl20535* silencing slowed glucose consumption. Strains i*gl20535*-3 and i*gl20535*-4 exhibited maximal biomass levels of 8.15 ± 0.04 g/L and 7.71 ± 0.12 g/L on day 9, representing decreases of 10.28% and 15.07%, respectively, compared to those of the WT strain. These results indicate that GL20535 influenced the rate of carbon source consumption in the medium but had limited impact on biomass accumulation, except for reducing a little biomass accumulation in the silenced strains during the late fermentation phase.

### 3.3. Effect of GL20535 on Polysaccharide Production and Monosaccharide Composition

#### 3.3.1. IPS

**Figure 5:**
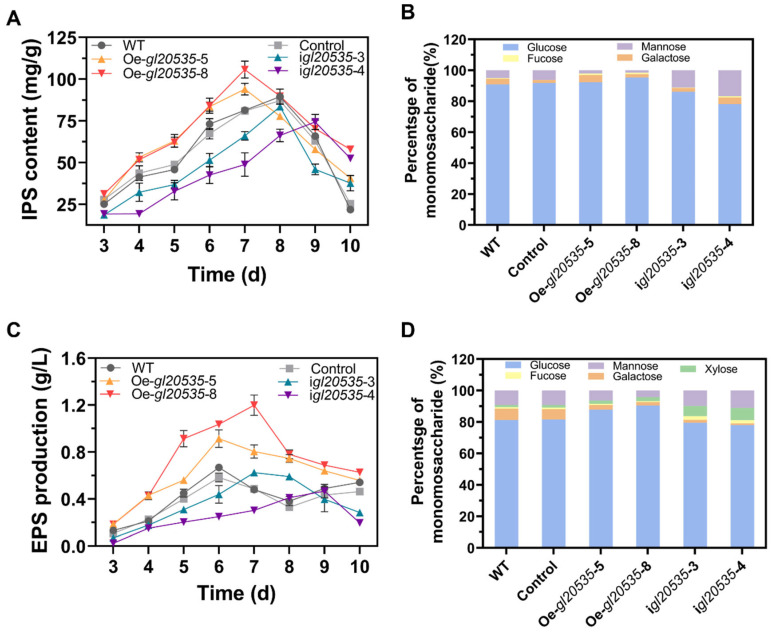
Effect of *gl20535* on polysaccharide production and monosaccharide composition. (**A**) IPS content. (**B**) Monosaccharide composition of IPS. (**C**) EPS production. (**D**) Monosaccharide composition of EPS. Results are expressed as mean ± standard deviation from triplicate experiments (n = 3).

The expression of *gl20535* not only affected mycelial morphology but also modulated polysaccharide synthesis in *G. lucidum*. Upregulation of *gl20535* accelerated IPS accumulation, manifested as maximum yields of Oe-*gl20535*-5 and Oe-*gl20535*-8 strains of 94.01 ± 3.08 mg/g and 105.75 ± 3.59 mg/g, representing an improvement of 4.97% and 18.08%, respectively, compared to those of the WT. IPS accumulation in the silenced strains was lower than that in the WT, with the maximum IPS content in the i*gl20535*-3 and i*gl20535*-4 strains decreasing by 6.77% and 16.97%, respectively (Figure 5A).

Additionally, the expression of *gl20535* in *G. lucidum* modified the monosaccharide composition of IPS. Compared to that in the WT, the glucose percentage increased by 1.34% and 4.43% in the Oe-*gl20535*-5 and Oe-*gl20535*-8 strains, respectively, while the mannose percentage decreased by 2.97% and 3.53%, respectively. Conversely, downregulation of *gl20535* resulted in a decrease in glucose percentage and an increase in mannose percentage (Figure 5B). The structure of IPS is primarily composed of glucose and mannose, which may be regulated through metabolic pathways involved in the synthesis of UDP-glucose and GDP-mannose nucleotide sugar precursors.

#### 3.3.2. EPS

The accumulation of EPS in the medium was also influenced by GL20535 (Figure 5C). Overexpression of *gl20535* promoted EPS accumulation, with the maximum yields of the Oe-*gl20535*-5 and Oe-*gl20535*-8 strains reaching 0.91 ± 0.06 g/L and 1.20 ± 0.06 g/L, representing increases of 36.7% and 79.4%, respectively, compared to those in the WT. Conversely, the maximum EPS yields in the i*gl20535*-3 and i*gl20535*-4 strains were 0.61 ± 0.01 g/L and 0.47 ± 0.04 g/L, reflecting decreases of 7.9% and 30.2%, respectively, compared to those in the WT (Figure 5C).

For the monosaccharide composition of EPS, upregulation of *gl20535* increased the glucose percentage by 6.59–9.21% and decreased the mannose and galactose percentage by 2.89–4.98% and 4.26–5.04%, respectively. Downregulation of *gl20535* decreased the glucan and galactose percentages by 1.77–3.32% and 5.36–6.06%, respectively, and increased the mannose and xylose percentages by 0.52–1.82% and 5.15–6.44%, respectively (Figure 5D). Taken together, EPS production may be influenced by polysaccharide export processes, including the expression of GTs and glycoside hydrolases (GHs).

### 3.4. Effect of gl20535 on the Cell Wall

**Figure 6:**
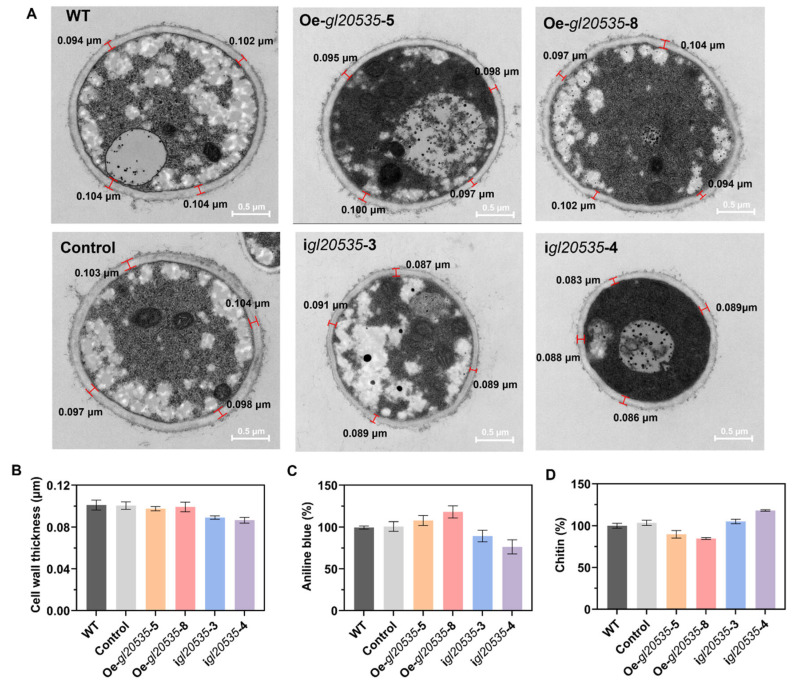
Effect of *gl20535* on the *G. lucidum* cell wall. (**A**) Electron microscopy of the cell wall. (**B**) Cell wall thickness. (**C**) Relative fluorescence value of *β*-1,3-glucan. (**D**) Chitin content. Results are expressed as mean ± standard deviation from triplicate experiments (n = 3).

The cell walls play critical roles in maintaining cell morphology and supporting growth in edible and medicinal fungi [38]. Mycelial cross-sections of *G. lucidum* recombinant strains were analyzed using a transmission electron microscope (Figure 6A). Overexpression of *gl20535* had no significant impact on cell wall thickness, with average values ranging from 0.098 μm to 0.101 μm (Figure 6B). In contrast, silencing *gl20535* resulted in a slight reduction in cell wall thickness (0.087–0.089 μm), although the difference was not statistically significant (Figure 6B).

*β*-1,3-glucan and chitin are the primary structural components of the *G. lucidum* cell wall, and their synthesis is catalyzed by *β*-1,3-glucosyltransferase and chitin synthase. Similar to intracellular and extracellular polysaccharides, *β*-1,3-glucan content in the cell walls of *gl20535* overexpression strains increased by 7.83–18.01% compared to that in the WT. Conversely, *gl20535* silencing reduced *β*-1,3-glucan content by 10.55–23.56% (Figure 6C). Meanwhile, chitin content in the cell wall decreased by 10.19–15.29% in overexpression strains, while it increased by 4.93–18.08% in silenced strains relative to those in the WT (Figure 6D). Overexpression of *gl20535* increased the *β*-1,3-glucan content in the cell wall and reduced chitin content by inhibiting chitin synthase expression, resulting in no significant change in cell wall thickness. In contrast, in the silenced strains, the changes in cell wall composition were reversed. The increased chitin could not compensate for the reduction in *β*-1,3-glucan, leading to a slight thinning of the cell wall. These results demonstrated that *gl20553* affects the contents of *β*-1,3-glucan and chitin in the cell wall and maintains the stability of the cell wall through co-regulation of their increase and decrease.

### 3.5. Transcript-Level Analysis of Genes in the Polysaccharide Biosynthesis Pathway

**Figure 7:**
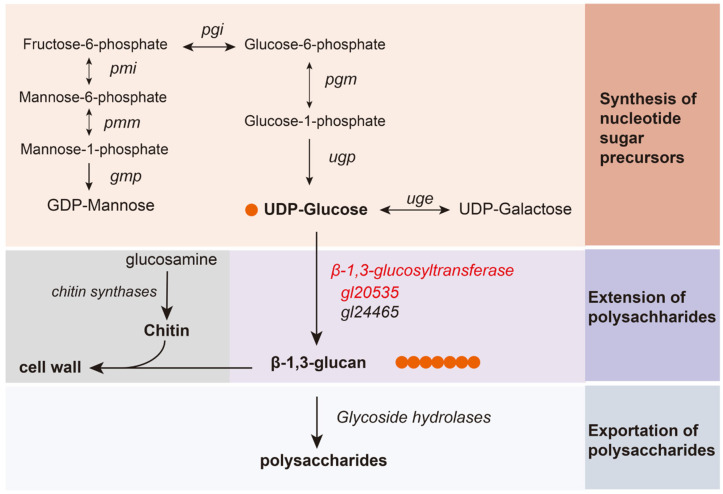
Key enzymes in polysaccharide synthesis pathway. GMP, GDP-mannose pyrophosphorylase; PGI, phosphoglucose isomerase; PGM, phosphoglucomutase; UGP, UDP-glucose pyrophosphorylase; PMM, phosphomannomutase; PMI, phosphomannose isomerase.

**Figure 8:**
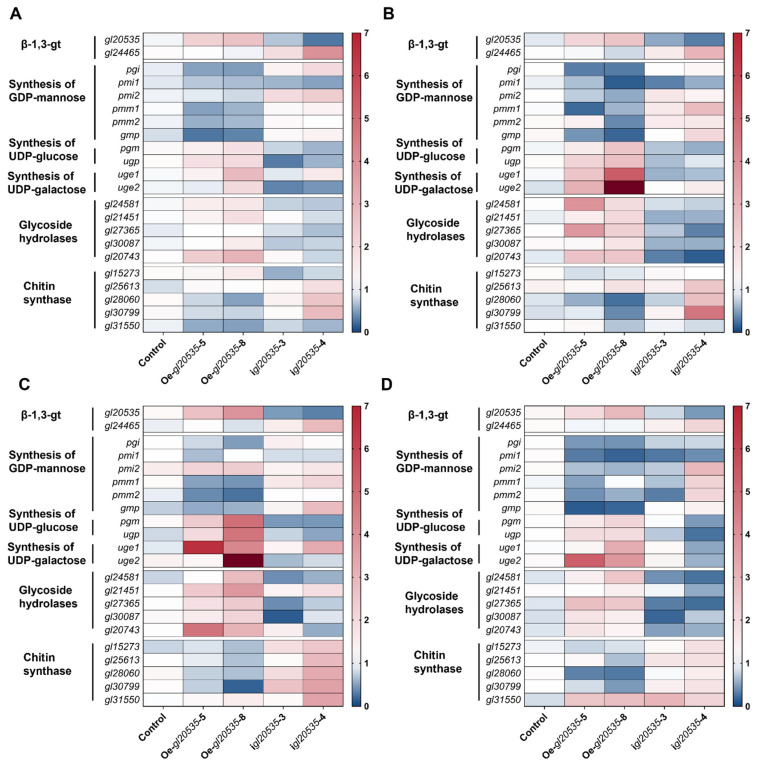
Transcript-level analysis of genes in the polysaccharide synthesis pathway during fermentation. (**A**) Day 4. (**B**) Day 5. (**C**) Day 6. (**D**) Day 7. The numbers on the color axis represent the relative expression levels of the genes.

To further elucidate the regulatory role of *β*-1,3-glucosyltransferase gene *gl20535* in polysaccharide biosynthesis, the transcript levels of associated pathway genes, including the *β*-1,3-glucosyltransferase isozyme gene *gl24465*, key genes in nucleotide sugar donor biosynthesis (*pgm*, *ugp*, *pgi*, *pmi*, *pmm*, *gmp*, *uge*), glycoside hydrolase genes (*gl24581*, *gl21451*, *gl27365*, *gl30087*, *gl20743*), and chitin synthase genes (*gl15273*, *gl25613*, *gl28060*, *gl30799*) were analyzed on days 4, 5, 6, and 7 of fermentation in recombinant strains (Figure 7 and Figure 8).

#### 3.5.1. *β*-1,3-glucosyltransferase Isozyme Gene *gl24465*

Overexpression of *gl20535* significantly elevated its transcript levels from day 4 to day 7, without notably affecting the expression of its isozyme gene *gl24465*. On day 6, *gl20535* expression peaked in strains Oe-*gl20535*-5 and Oe-*gl20535*-8, showing increases of 165.88% and 289.22%, respectively, over those in the WT, while *gl24465* expression remained unchanged (Figure 8C).

In contrast, silencing *gl20535* significantly upregulated *gl24465* expression, with the magnitude of compensation positively correlated with silencing efficiency. On day 6, strains i*gl20535*-3 and i*gl20535*-4 exhibited *gl20535* expression reductions of 54.48% and 67.89%, respectively, accompanied by *gl24465* upregulation of 51.04% and 186.79%, respectively (Figure 8C).

#### 3.5.2. Key Enzymes in Nucleotide Sugar Donor Biosynthesis

In the nucleotide sugar biosynthesis pathway, *gl20535* overexpression upregulated *pgm*, *ugp*, and *uge*, thereby promoting UDP-glucose biosynthesis. Simultaneously, it suppressed *pgi*, *pmi*, *pmm*, and *gmp*, which are involved in GDP-mannose biosynthesis. These changes suggest a metabolic flux shift favoring UDP-glucose production, consistent with the increased intracellular glucose content and reduced mannose and galactose proportions in the polysaccharide profile. Conversely, *gl20535* silencing reversed these trends (Figure 8). On day 7, strain i*gl20535*-3 exhibited an overall downregulation of genes involved in sugar donor biosynthesis (Figure 8D).

#### 3.5.3. Glycoside Hydrolases

Glycoside hydrolases are involved in the modification and export of polysaccharides, thereby influencing exopolysaccharide accumulation [20]. In *gl20535*-overexpressing strains, glycoside hydrolase genes were broadly upregulated, particularly on day 6, with strain Oe-*gl20535*-8 showing the highest transcript levels. In contrast, *gl20535* silencing downregulated these genes, with i*gl20535*-4 exhibiting the most pronounced reduction. These results indicated a positive correlation between *gl20535* expression and glycoside hydrolase activity (Figure 8).

#### 3.5.4. Chitin Synthases

Chitin, a major component of the cell wall, is synthesized by multiple chitin synthases regulated by *gl20535*. Overexpression of *gl20535* resulted in the progressive downregulation of chitin synthase genes during fermentation (Figure 8), contributing to reduced chitin content and unchanged cell wall thickness (Figure 6). Conversely, silencing *gl20535* enhanced chitin synthase expression, with the most significant upregulation observed on day 6 (Figure 8), contributing to increased chitin content and reduced cell wall thickness (Figure 6).

## 4. Discussion

GLPs are widely recognized for their pharmacological potential and have become a focal point in the development of functional foods [39,40]. However, their complex biosynthetic pathways pose substantial challenges for large-scale application in the food industry [7]. Among the enzymes involved in GLP synthesis, *β*-1,3-glucosyltransferase plays a central role in the elongation of glucan chains [20], while the underlying metabolic regulation remains insufficiently understood [19,21,41]. In this study, we elucidated the mechanism and function of GL20535 in *G. lucidum*.

Previous studies have found that the interaction patterns between *β*-1,3-glucosyltransferase isozymes differ across fungal species [33]. In *Saccharomyces cerevisiae* [34] and *Candida albicans* [35], FKS1 is indispensable and functionally dominant, while other isozymes lack compensatory capacity. In contrast, all *β*-1,3-glucosyltransferase isoforms in *Schizosaccharomyces pombe* are functionally non-redundant [42]. In this study, *gl24465* expression remained unchanged in the *gl20535*-overexpressing strains, consistent with the findings in *G. lingzhi* [21]. Silencing *gl20535* induced a compensatory upregulation of *gl24465*, suggesting a potential supplemental mechanism in *G. lucidum*. The functional diversity of *β*-1,3-glucosyltransferases is also evident in other edible and medicinal fungi. For instance, silencing *GFGLS2* in *G. frondosa* significantly reduces polysaccharide synthesis, with no compensatory effect observed from its isoenzymes [19]. In *Agaricus bisporus*, the expression patterns of isozymes are largely determined by the developmental stages [43]. These phenomena suggest that *β*-1,3-glucosyltransferase exhibits species-specific regulation, as observed in *Ascomycota* and *Basidiomycota*.

In the sugar donor synthesis pathway, *gl20535* influences the expression of key genes. Its overexpression upregulates *pgm* and *ugp*, thereby enhancing UDP-glucose biosynthesis. This likely reflects a feedback mechanism in which increased consumption of UDP-glucose for sugar chain extension stimulates metabolic flux. Notably, previous studies have shown that overexpression of *pgm* [10,44] and *ugp* [9,45] can enhance *β*-1,3-glucosyltransferase expression levels, suggesting a feedback regulation between *β*-1,3-glucosyltransferase and UDP-glucose synthesis. Similar feedback mechanisms have been documented in plant starch and cellulose metabolism [46]. Furthermore, overexpression of *gl20535* suppressed the expression of *pmi*, *pmm*, and *gmp*, thereby shifting the carbon flux toward UDP-glucose, and increased glucose proportion in GLPs. According to genome-scale metabolic network model analysis, the activities of *pgm* and *pgi* can regulate the flow of glucose-6-phosphate to GDP-mannose or UDP-glucose synthesis, resulting in changes in monosaccharide composition [8,47]. This is consistent with previous findings in *G. frondosa*, in which *ugp* silencing upregulated *gmp* [44]. In contrast, *gl20535* silencing induced the opposite effect. Interestingly, in other edible and medicinal fungi, such as *G. lingzhi* [21,22] and *G. frondosa* [17,19], *β*-1,3-glucosyltransferases showed limited impact on sugar donor biosynthesis, reflecting their specificity among different species.

GHs contribute to polysaccharide modification and export by catalyzing the hydrolysis of glycosidic bonds. In *gl20535*-overexpressing strains, multiple GH genes (*gl21451*, *gl24581*, *gl30087*, *gl24039*, and *gl27365*) were significantly upregulated, consistent with the higher EPS production. This transcriptional activation was consistent with the observed increase in polysaccharide yield, particularly the more pronounced elevation of EPS compared to IPS. These enzymes were also highly expressed in high polysaccharide-producing *G. lucidum* strains, underscoring their contribution to EPS accumulation [20].

Our findings indicate that *gl20535* coordinates multiple layers of the polysaccharide biosynthesis pathway, from sugar precursor synthesis to polysaccharide hydrolysis, and regulates GLP production without mycelial biomass. Overexpression of *gl20535* significantly enhanced polysaccharide yield, echoing the results of similar overexpression strategies in *Pleurotus ostreatus* [41] and *G. lingzhi* [21,22]. Conversely, its silencing led to a marked reduction in yield, similar to observations in *G. frondosa* [17,19]. Collectively, these results highlight *β*-1,3-glucosyltransferase as a key regulatory enzyme in polysaccharide production in edible and medicinal fungi.

GTs also influence the cell wall due to their involvement in cell wall polysaccharide synthesis. For instance, in *G. frondosa*, silencing *β*-1,3-glucosyltransferase genes led to thinner mycelium [17,19], and overexpression of glucosyltransferase gene *UGT88A1* increased sensitivity to cell wall stress [18]. In *G. lucidum*, overexpression of *α*-glucosyltransferase reduced *β*-1,3-glucan content in the cell wall without altering chitin levels [16]. Unlike previous reports, GL20535 influenced chitin content by regulating the expression of chitin synthase genes in response to changes in *β*-1,3-glucan levels, resulting in no significant alteration in cell wall thickness. These findings elucidate the differential effects of glucosyltransferases on cell wall synthesis in edible and medicinal fungi.

The regulatory mechanism of GL20535 in GLP biosynthesis has been basically clarified, while the interaction and feedback mechanisms of its isozyme (GL24465) require further investigations. This will facilitate a comprehensive elucidation of the mechanism of action of *β*-1,3-glucosyltransferase and enhance the in-depth understanding of the polysaccharide biosynthesis process.

## 5. Conclusions

*β*-1,3-glucosyltransferase (GL20535) plays an important role in polysaccharide synthesis. GL20535 influences the synthesis of UDP-glucose and GDP-mannose in the sugar donor biosynthesis pathway, thereby enhancing polysaccharide yield and altering monosaccharide composition. During sugar chain extension, *β*-1,3-glucosyltransferase isozyme gene *gl24465* exhibited compensatory upregulation in response to *gl20535* silencing. Furthermore, *gl20535* regulated the transcription of several glycoside hydrolase genes (*gl24681*, *gl27365*, *gl30087*, and *gl20743*) associated with EPS accumulation. These findings highlight the functional role of GL20535 in *G. lucidum* and promote the application of GLPs in the food production industry.

## Data Availability

Data will be made available on request.

## Supplementary Materials

The following supporting information can be downloaded at: https://www.mdpi.com/article/10.3390/jof11070532/s1, Figure S1: Construction of the *gl20535*-silencing plasmid pMsdhB-i*gl20535* and *gl20535*-overexpression plasmid pMsdhB-OE*gl20535*; Figure S2: Ion chromatogram of product separation. (A) The standard solution contains fucose, galactose, glucose, xylose and mannose. (B) Ion chromatogram of IPS samples. (C) Ion chromatogram of EPS samples; Figures S3–S14: Full, uncropped TEM images; Table S1: Primers used to clone genes; Table S2: Primers used to RT-qPCR.

## Abbreviations

EPS, extracellular polysaccharides; F-6-P, fructose-6-phosphate; GLPs, *Ganoderma lucidum* polysaccharides; GT, glucosyltransferase; GH, glycoside hydrolases; G-6-P, glucose-6-phosphate; G-1-P, glucose-1-phosphate; GMP, GDP-mannose pyrophosphorylase; IPS, intracellular polysaccharides; M-1-P, mannose-1-phophate; M-6-P, mannose-6-phosphate; PGI, phosphoglucose isomerase; PGM, phosphoglucomutase; UGP, UDP-glucose pyrophosphorylase; PMM, phosphomannomutase; PMI, phosphomannose isomerase.
