# Supplementary material for: Mechanism Underlying Ganoderma lucidum Polysaccharide Biosynthesis Regulation by the β-1,3-Glucosyltransferase Gene gl20535
Source: J Fungi (Basel). 2025 Jul 17;11(7):532. doi: 10.3390/jof11070532 (PMC12295571; doi:10.3390/jof11070532)
Supplement: Supplementary file 1 [file jof-11-00532-s001.zip › jof-3738654-supplementary.pdf]

Supplementary Material

Mechanism underlying *Ganoderma lucidum* polysaccharide biosynthesis regulation by the β-1,3-glucosyltransferase gene *gl20535*

Jingyun Liu[a,b,1], Mengmeng Xu[a,b,d], Mengye Shen[a,b], Junxun Li[c,*], Lei Chen[a,b], Zhenghua Gu[a,b], Guiyang Shi[a,b], Zhongyang Ding[a,b,*]

[a] School of Biotechnology and Key Laboratory of Carbohydrate Chemistry and Biotechnology of Ministry of Education, Jiangnan University, Wuxi 214122, China

[b] National Engineering Research Center for Cereal Fermentation and Food Biomanufacturing, Jiangnan University, Wuxi 214122, China

[c] Shandong Taishan Shengliyuan Group Co. Ltd., Shandong 271000, China

[d] School of Food Science and Technology, Jiangnan University, Wuxi 214122, China

*Corresponding author at: Shandong Taishan Shengliyuan Group Co. Ltd., Shandong 271000, China. E-mail address: li_junxun@sina.com (J. Li)

*Corresponding author at: Key Laboratory of Carbohydrate Chemistry and Biotechnology, Ministry of Education, School of Biotechnology, Jiangnan University, Wuxi 214122, China. E-mail address: zyding@jiangnan.edu.cn; bioding@163.com (Z. Ding).

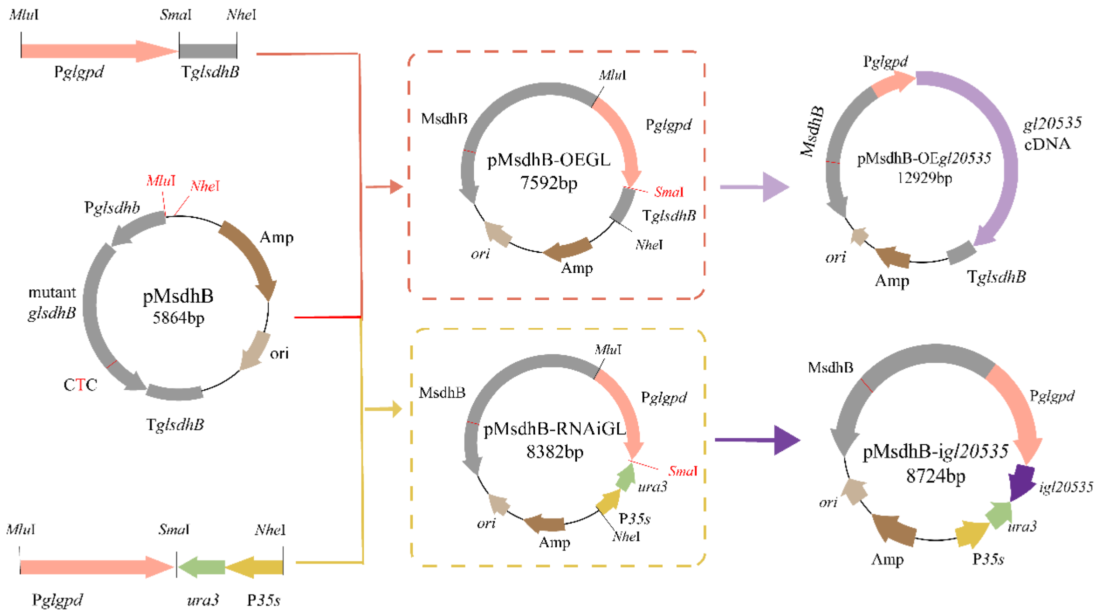

Figure S1. Construction of the *gl20535*-silencing plasmid pMsdhB-i*gl20535* and *gl20535*-overexpression plasmid pMsdhB-OE*gl20535*

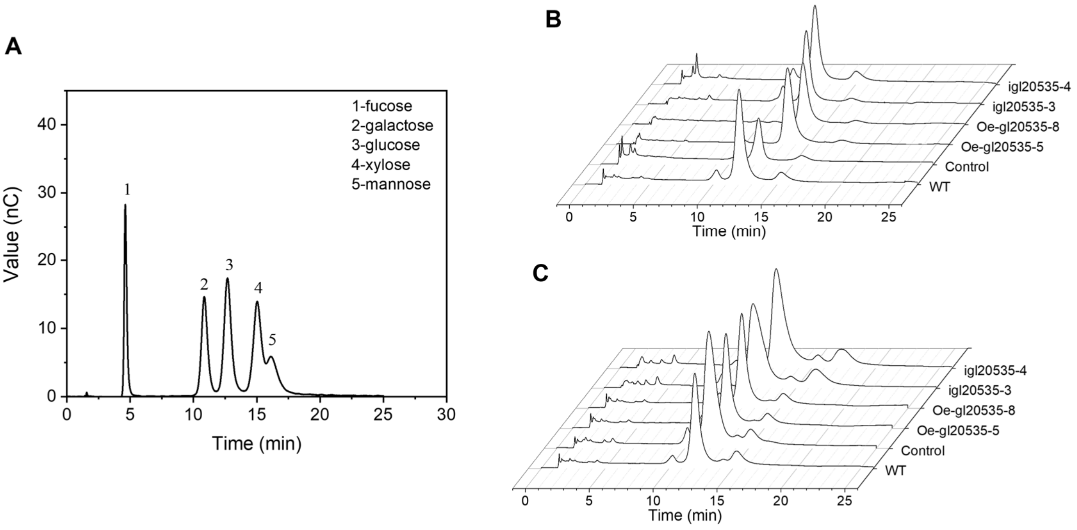

Figure S2. Ion chromatogram of product separation.

(A) The standard solution contains fucose, galactose, glucose, xylose and mannose. (B) Ion chromatogram of IPS samples. (C) Ion chromatogram of EPS samples.

Table S1. Primers used to clone genes

Primer	Sequences (5'to3')	Genes for cloned
gl20535-F	ATGGCCTCCCGCCATGGCCACCACA	*gl20535*
gl20535-R	TCAGATGCCCTGGCAAATGCTGCAG	
glsdhB-F	GCTAGCAATTACCACGCGTTCTGCTCTTCCCGATTGCTG	*glsdhB*
glsdhB-R	CTATGTCTTGCCTTGTCTCGC	
M*sdhB*-F	CCTATACCGCTGCCTCACGATCTTCAACTG	M*sdhB*
M*sdhB*-R	CAGTTGAAGATCGTGAGGCAGCGGTATAGG	
P*gpd*-F	TCCAAAGCCGCTCTCATGGC	P*glgpd*
P*gpd*-R	CTCTGACCCGCTCATCCCGGGGACCTTGACCTGT	
T*sdhB*-F	TCACAGGTCAAGGTCCCCGGGATGAGCGGGTCAGAG	T*glsdhB*
T*sdhB*-R	CGGATCTTCCAGAGAGCTAGCTGCTCTATGTCTTGC	
ura3-F	CATCCCCCTCTCAACCCCGGGGGTACCTCTCCGCCT	*ura3*
ura3-R	GAGAGGACACGCTGAGGTGGCACTTCAATA	
P*35s*-F	TATTGAAGTGCCACCTCAGCGTGTCCTCTC	P*35s*
P*35s*-R	TGAGACTTTTCAACAAAGGG	

Table S2. Primers used to RT-qPCR

Primers	Sequences (5' to 3')	RT-qPCR for genes
18S-qpcr-F	TATCGAGTTCTGACTGGGTTGT	housekeeping gene
18S-qpcr-R	ATCCGTTGCTGAAAGTTGTAT	
gl20535-qpcr-F	CATGCTGCTGTATGTGACGC	*gl20535*
gl20535-qpcr-R	GAATAAGAACGGCGACACGC	
pgi-qpcr-F	GCACAGACCCACGCACTCCT	phosphoglucose isomerase gene
pgi-qpcr-R	TAACACGCTGCCCGCCCTTG	
pmi1-qpcr-F	CTCGTCTTTGAAGCGGTCCAC	phosphomannose isomerase gene
pmi1-qpcr-R	CATACTTCCCCATCGCGTCT	
pmi2-qpcr-F	GGCTTGACTAAGGGCCAAGA	
pmi2-qpcr-R	TCGACTCGGATTTCGTTCCC	
pmm1-qpcr-F	CCTAACGGGTGGGACAAGAC	Phosphomannomutase gene
pmm1-qpcr-R	TCTCCTCGAATTGCTCGTCGG	
pmm2-qpcr-F	AAGAAGGTTGTGATCGGCGT	
pmm2-qpcr-R	GTCGATGACTCTCGAGCCTG	
gmp-qpcr-F	GAGGGTCTGAGTCACAACGG	GDP-mannose pyrophosphorylase gene
gmp-qpcr-R	CCAGGGACGGGATTGAGTTC	
pgm-qpcr-F	GTACAACATGTCCAACGGCG	Phosphoglucomutase gene
pgm-qpcr-R	GGGCTAGTTCGATGACCCTG	
ugp-qpcr-F	TCGACACCGAGATGCAGTCG	UDP-glucose pyrophosphorylase gene
ugp-qpcr-R	CGAGACCACCATTGACCTTC	
uge1-qpcr-F	TCGGAACTCAAACGAGTGCT	UDP-glucose-4-epimerase gene
uge1-qpcr-R	CTTGTATCTCCGCGTCTGCT	
uge2-qpcr-F	GTCGCGTTGCTCGCACGTCA	
uge2-qpcr-R	CCTTGCCGTACTTTGCGAAC	
gl24465-qpcr-F	GTGTGATCGCCATCATTGCC	β-1,3-glucosyltransferase gene *gl24465*
gl24465-qpcr-R	ACCACGACCGTACCACTTTC	
gl24554-qpcr-F	CACGAACGTCACGGACCAT	α-1,3-glucosyltransferase *gl24554*

gl24554-qpcr-R	ACGTGTACGTGTTACCCGAG	
gl24971-qpcr-F	TTCCAGAACCTGCACTCGAC	α-1,4-glucosyltransferase gene
gl24971-qpcr-R	GTACTCATACCGACCAGCGG	*gl24971*
gl22535-qpcr-F	AAGCACAAGGAGGAGACTGC	β-1,6-glucosyltransferase gene
gl22535-qpcr-R	CTCTTCGTGTGACGGAGGAC	*gl22535*
gl30087-qpcr-F	TGGCATTCCAACCAGACGAA	
gl30087-qpcr-R	ACAGCGGAGAGAACATTGGG	
gl24581-qpcr-F	CGAAGCGACACTACTTGGGT	
gl24581-qpcr-R	CACTTGGGATGAACGGGTGA	
gl21451-qpcr-F	CTCAGCTTGGGCGGTCAATA	Glycoside hydrolase gene
gl21451-qpcr-R	AGGTCTGGGCGTAATTGGTG	
gl20743-qpcr-F	GACCCTCGCAATGGACAAGA	
gl20743-qpcr-R	GCCGTAGAAGAAGTCTCCCG	
gl27365-qpcr-F	CCTGACCCTTCTGTCGCTTT	
gl27365-qpcr-R	CACGGATGCCAATTTGGGTG	
gl15273-qpcr-F	TGTTCGAGTCACACGTCCAG	
gl15273-qpcr-R	AGGCGAACACATTCCGAGTT	
gl25613-qpcr-F	GGTGAACGTAAGGGCGAAG	
gl25613-qpcr-R	GTCGTGAACGTTCCCAGTCT	
gl28060-qpcr-F	TGTTCGAGTACACCACGACG	Chitin synthase gene
gl28060-qpcr-R	AAGAACCACCGATGCGAGTT	
gl30799-qpcr-F	GGAACCCGCTCGAGGTTAAA	
gl30799-qpcr-R	TGGAGGAACGCCATGAGAAC	
gl31550-qpcr-F	CATTCTGGATTGCGCCTTCG	
gl31550-qpcr-R	AAGAGACTTGTGCCTGGACG	

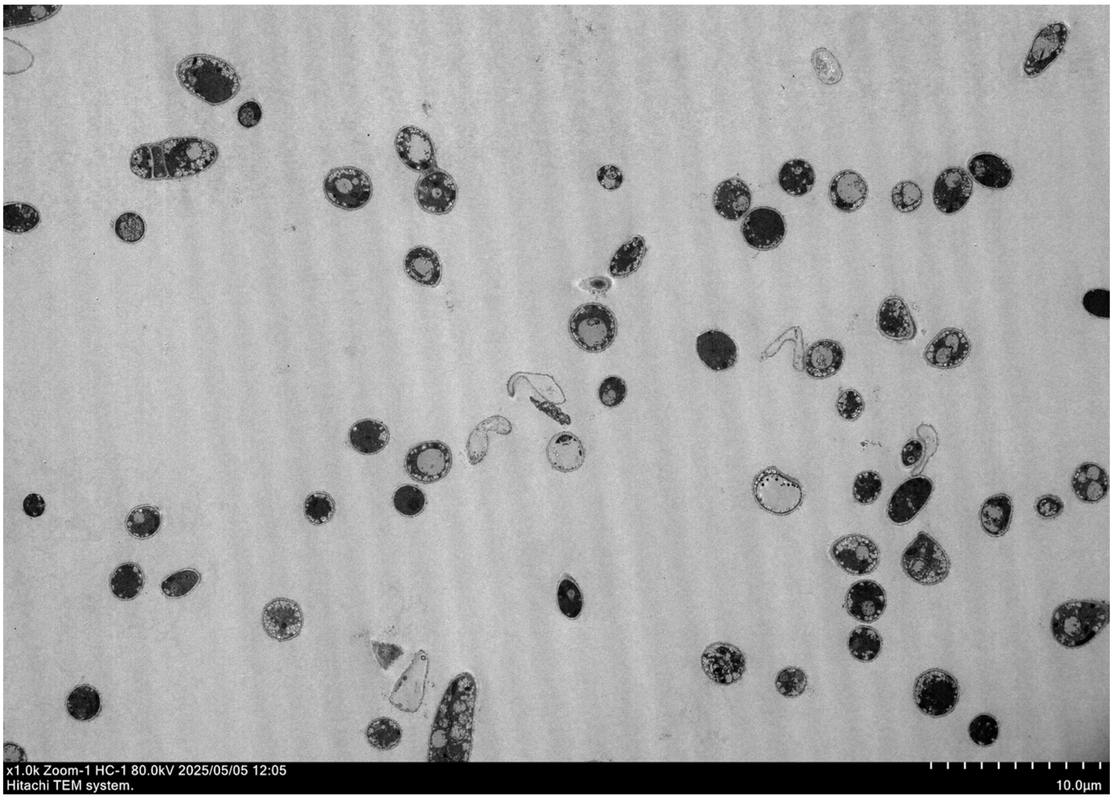

Figure S3. Full, uncropped TEM images of WT mycelium in Figure 3.

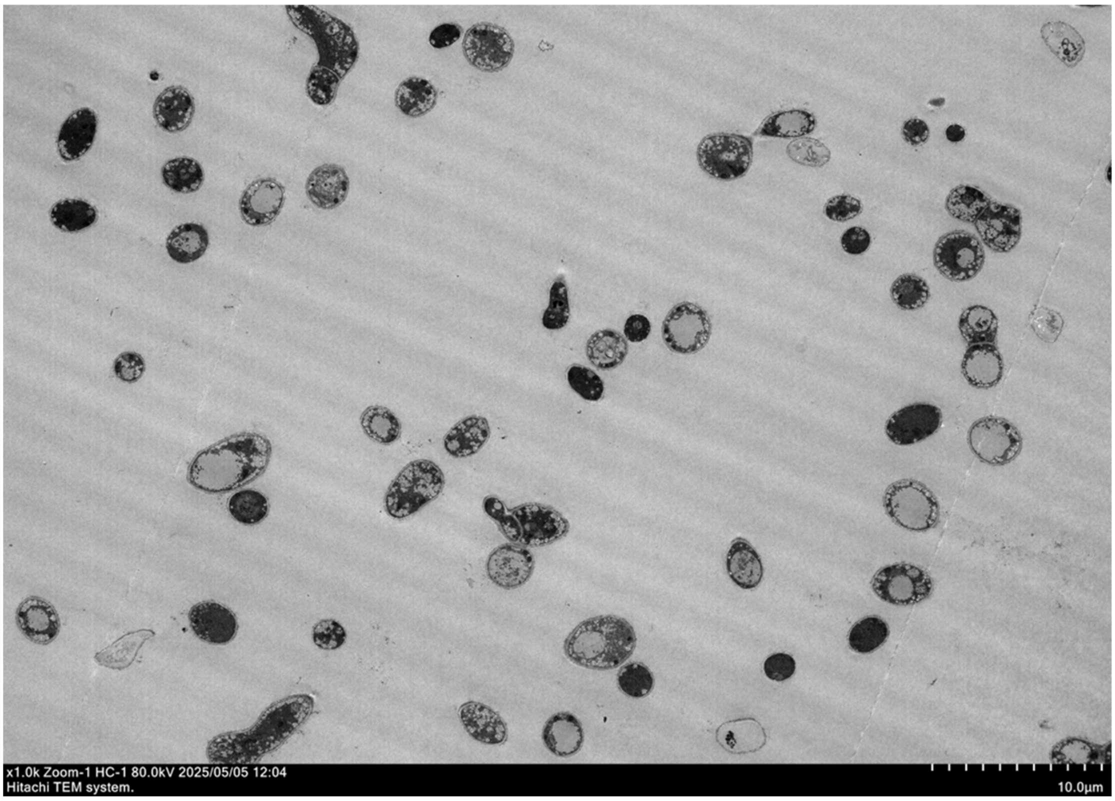

Figure S4. Full, uncropped TEM images of Control mycelium in Figure 3.

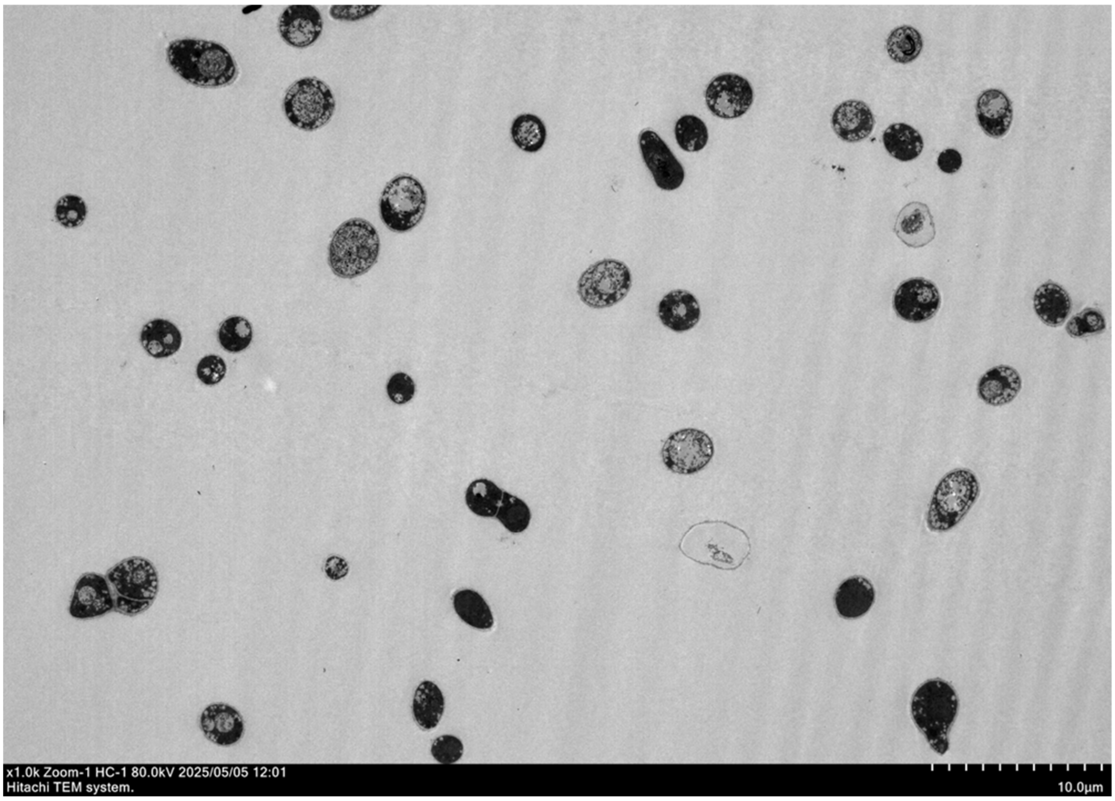

Figure S5. Full, uncropped TEM images of Oe-*gl20535*-5 mycelium in Figure 3.

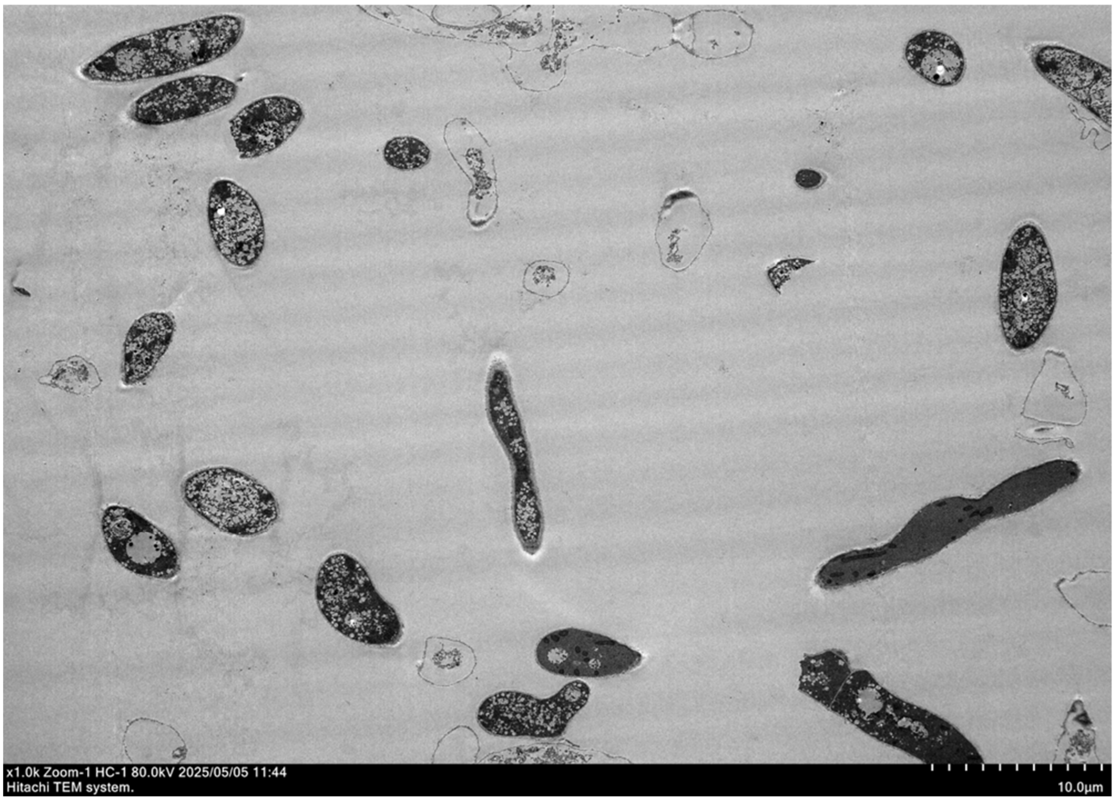

Figure S6. Full, uncropped TEM images of Oe-*gl20535*-8 mycelium in Figure 3.

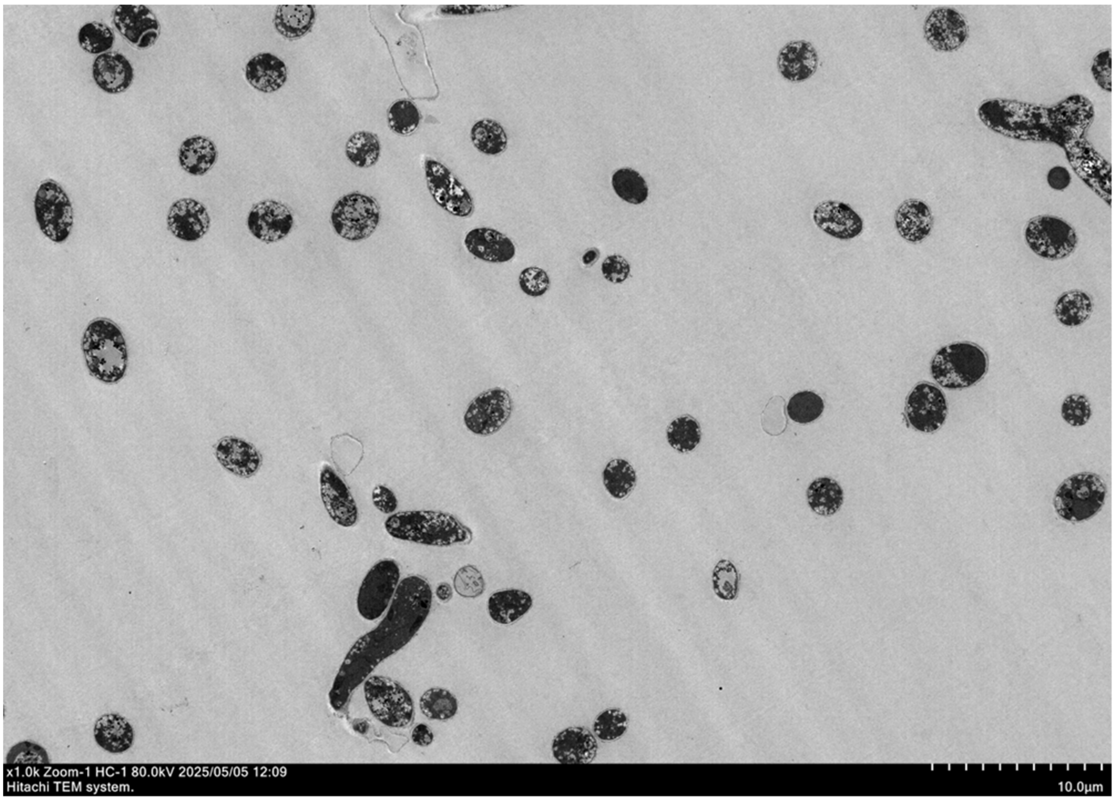

Figure S7. Full, uncropped TEM images of i*gl20535*-3 mycelium in Figure 3.

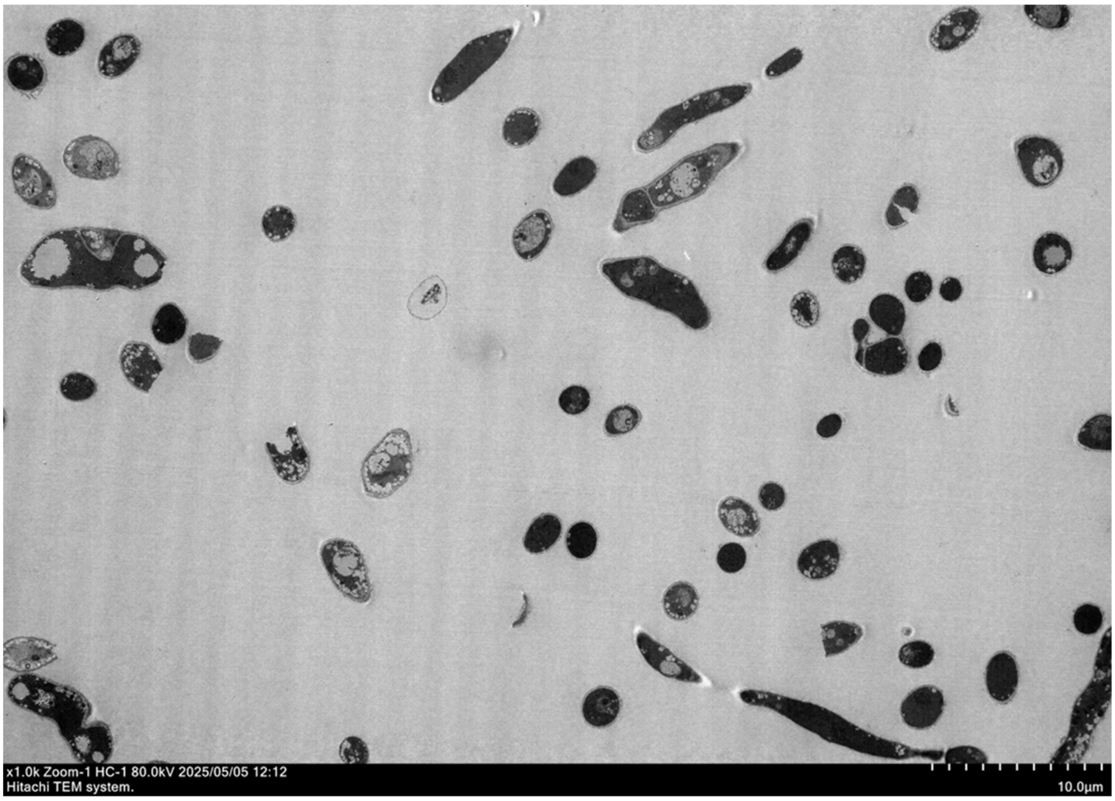

Figure S8. Full, uncropped TEM images of i*gl20535*-4 mycelium in Figure 3.

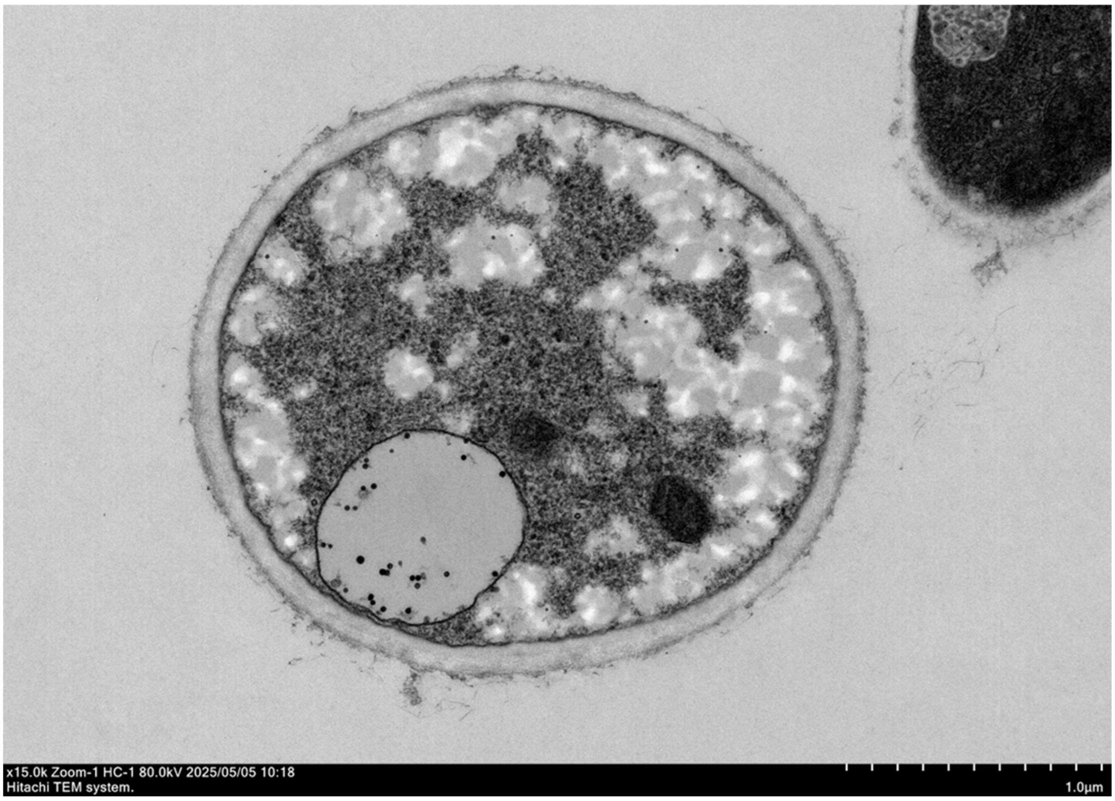

Figure S9. Full, uncropped TEM images of WT mycelium in Figure 6.

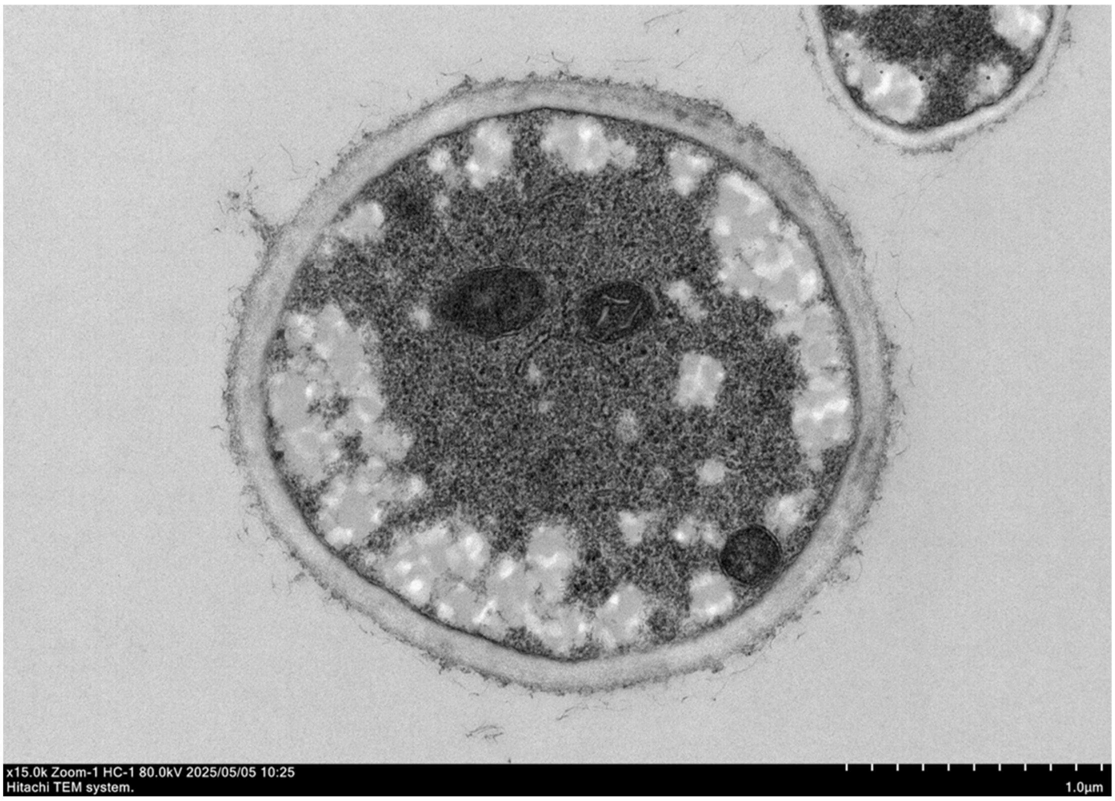

Figure S10. Full, uncropped TEM images of Control mycelium in Figure 6.

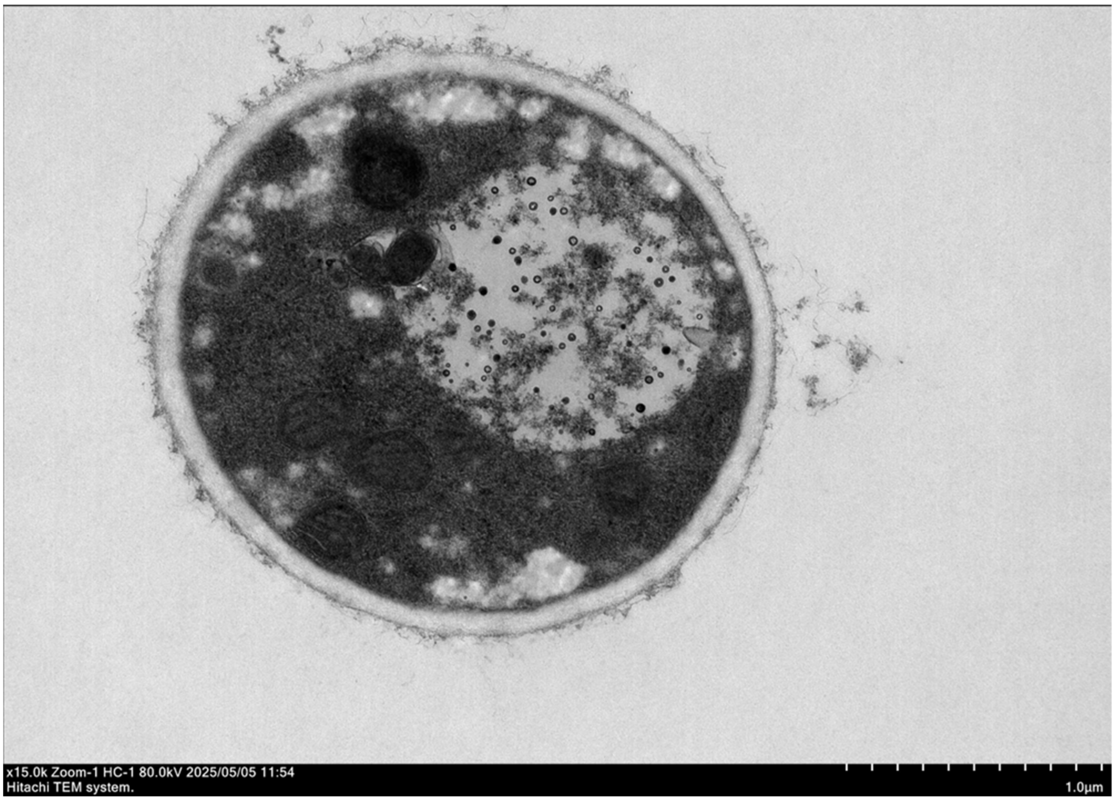

Figure S11. Full, uncropped TEM images of Oe-*gl20535*-5 mycelium in Figure 6.

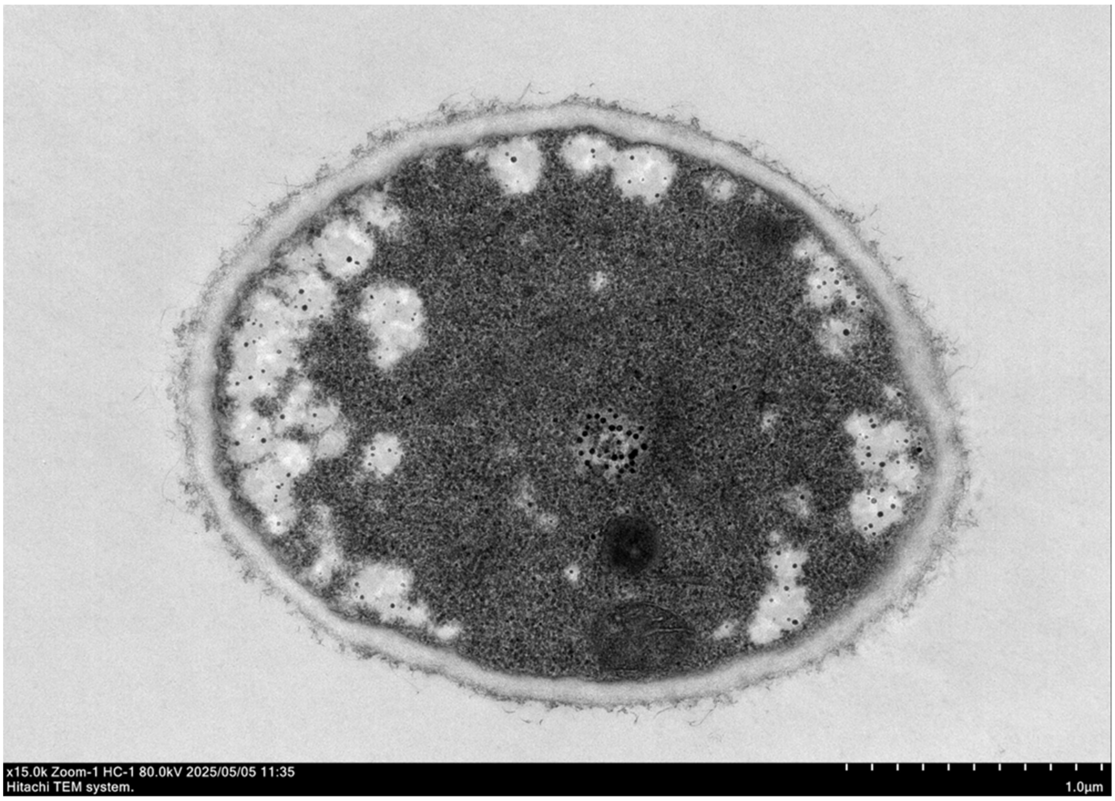

Figure S12. Full, uncropped TEM images of Oe-*gl20535*-8 mycelium in Figure 6.

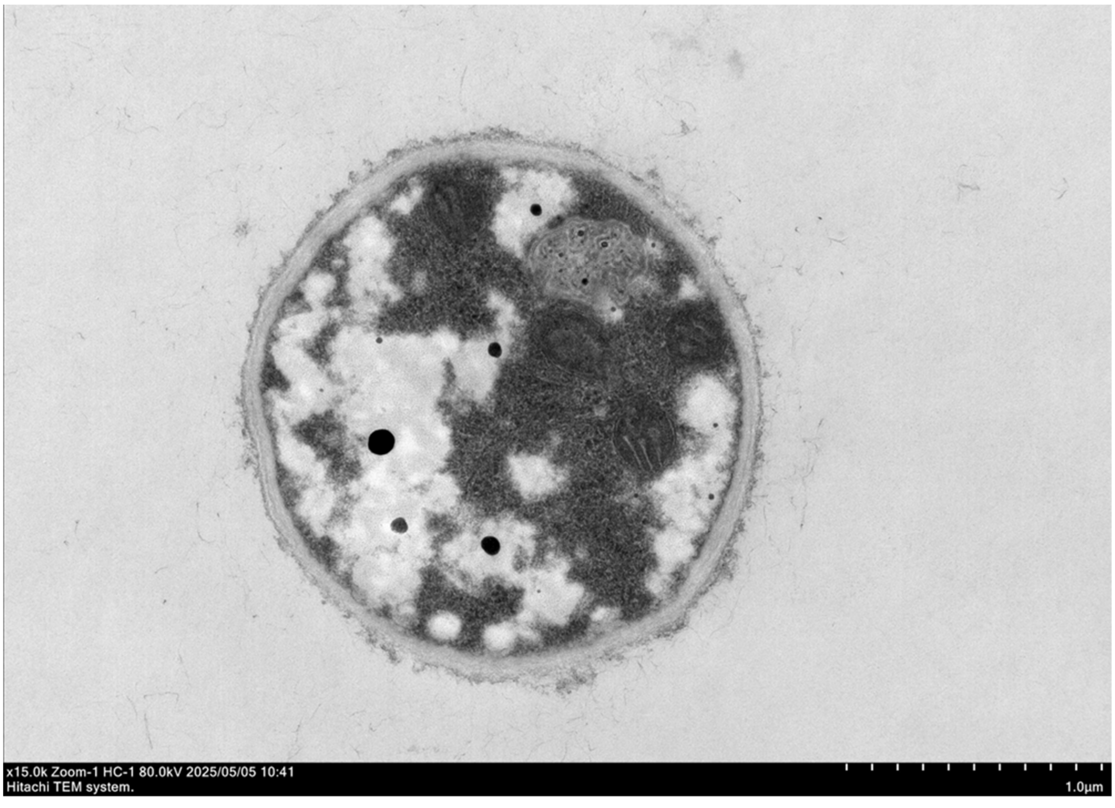

Figure S13. Full, uncropped TEM images of i*gl20535*-3 mycelium in Figure 6.

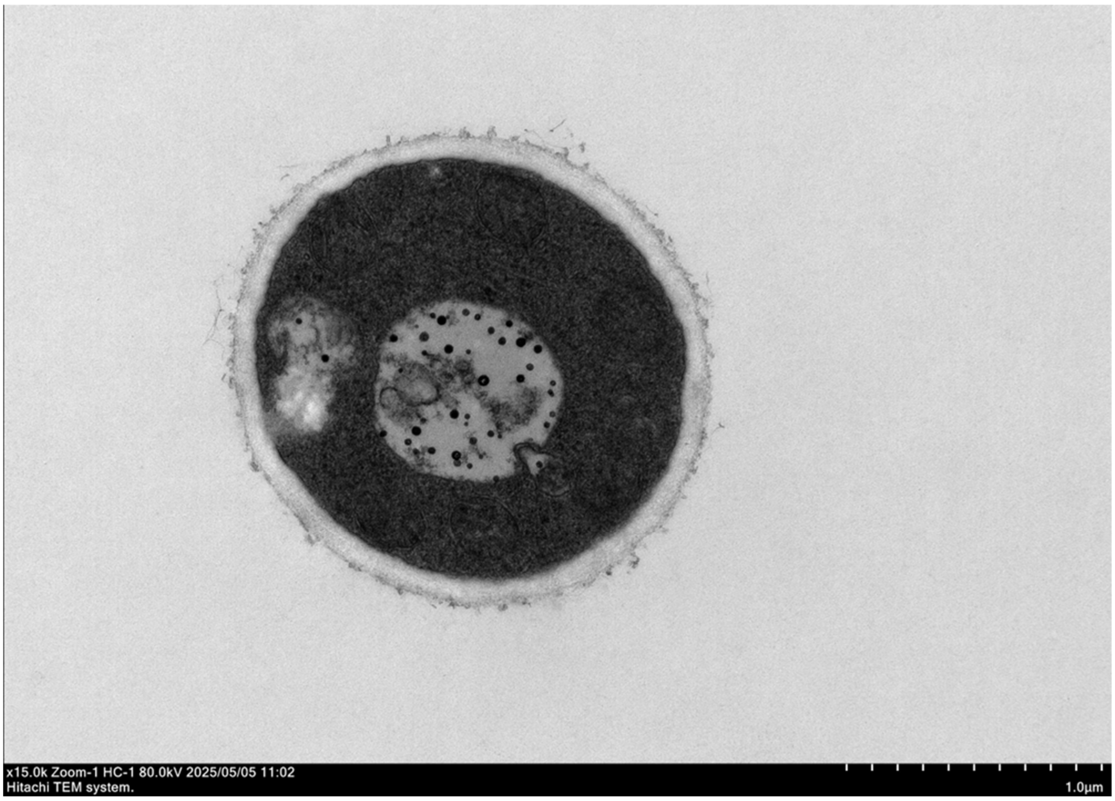

Figure S14. Full, uncropped TEM images of i*gl20535*-4 mycelium in Figure 6.
